# Shifting Valproic Acid to Levetiracetam in Women of Childbearing Age With Epilepsy: A Retrospective Investigation and Review of the Literature

**DOI:** 10.3389/fneur.2020.00330

**Published:** 2020-04-24

**Authors:** Cheng-Yen Kuo, Yi-Hsuan Liu, I-Jun Chou, Huei-Shyong Wang, Po-Cheng Hung, Min-Liang Chou, Jainn-Jim Lin, Shih-Yun Lan, Meng-Ying Hsieh, Yi-Shan Wang, Kuang-Lin Lin

**Affiliations:** ^1^Department of Pediatrics, Chang Gung Children's Hospital and Chang Gung Memorial Hospital, Taoyuan, Taiwan; ^2^Division of Pediatric Neurology, Chang Gung Children's Hospital and Chang Gung Memorial Hospital, Taoyuan, Taiwan; ^3^College of Medicine, Chang Gung University, Taoyuan, Taiwan; ^4^Division of Pediatric Critical Care and Pediatric Neurocritical Care Center, Chang Gung Children's Hospital and Chang Gung Memorial Hospital, Taoyuan, Taiwan; ^5^Department of Pediatric Neurology, Saint Paul's Hospital, Taoyuan, Taiwan

**Keywords:** women with epilepsy, major congenital malformation, valproic acid, levetiracetam, switching, pregnancy

## Abstract

**Objective:** Valproic acid is the most high-risk teratogenic antiepileptic drug, and it may lead to fetal major congenital malformations. However, it is still used in women of childbearing age with epilepsy. The aim of this study was to report our experience of discontinuing or lowering valproic acid by adding levetiracetam, a low-risk teratogenic antiepileptic drug.

**Methods:** We reviewed the medical records of childbearing age female patients with epilepsy who were treated with valproic acid initially and then switched to levetiracetam. The clinical profiles were recorded. The primary outcome was successful switching, which was defined as a decrease in the daily valproic acid dosage, after levetiracetam had been added.

**Results:** Twenty-four female patients were enrolled (median age 22 years). The successful switching rate was 83.3% (20/24), and 55% (11/20) discontinued valproic acid after levetiracetam had been added. There were no significant differences between the successful and unsuccessful groups in etiology, electroencephalogram, and magnetic resonance imaging findings. Pharmacoresistant to levetiracetam was much higher in the unsuccessful group (45 vs. 100%). The median switching duration was 19.5 months in the successful group. There were improvements in metrorrhagia and alopecia in all of the patients in the successful group after valproic acid had been tapered.

**Conclusions:** Our experience supports switching valproic acid to levetiracetam in childbearing age women with epilepsy as an effective strategy to lower the teratogenic rate and adverse effects. A long switching period was noted in this study. We suggest starting early in childbearing age women with epilepsy.

## Introduction

Seizure control and antiepileptic drug-related fetal congenital malformations, cognitive impairment and autism spectrum are concerns for women with epilepsy (WWE) when facing pregnancy. A cross-sectional study in the USA reported that half of all pregnancies are unplanned (1). When facing this situation, some women choose to stop taking antiepileptic drugs abruptly due to fears of related fetal effects. According to data from EURAP (European and International Registry of Antiepileptic Drugs in Pregnancy), discontinuing or switching antiepileptic drugs during pregnancy may lead to further seizures compared to those who continue treatment (2). The key predictive factor has been reported to be good seizure control in the year prior to pregnancy, and the risk of seizures during pregnancy has been reported to be 3–4 times higher in women who have seizures in the year prior to pregnancy (3). Planning for pregnancy with good seizure control using low-risk teratogenic antiepileptic drugs and avoiding unplanned pregnancies are the most important issues in WWE. However, how best to plan and manage with high-risk teratogenic drugs has yet to be determined.

Meadow et al. first reported an association between antiepileptic drugs and congenital malformations (4). The first pregnancy registry, International Lamotrigine Pregnancy Registry (5), was established in 1992, and there are currently six national and international pregnancy registries (5–10). According to these studies, antiepileptic drugs can be classified as having a high or low risk of teratogenicity (11). Valproic acid (VPA) is classified as being a high-risk for teratogenicity (9), and it has been associated with increased rates of offspring cognitive impairment and autism disorders (12–16). Moreover, both congenital malformations and cognitive impairment have been reported to be dose dependent (12, 17, 18). Levetiracetam, a new antiepileptic drug with a low-risk for teratogenicity (19), has been proven to be effective for juvenile myoclonic epilepsy (20), and to have a similar efficacy in the treatment of new-onset epilepsy to VPA (21). Its use has also been suggested in pregnancy (22). In a literature review, no published articles were found about the success rate of shifting antiepileptic drugs in WWE planning pregnancy. Therefore, the purpose of this article was to share our experience in shifting VPA to levetiracetam in WWE of childbearing age.

## Patients and Methods

We retrospectively reviewed the medical records of WWE who were treated with VPA initially and then shifted to levetiracetam at Chang Gung Children's Hospital, Linkou Branch from January 2002 to December 2018. Women who were not within childbearing age, had poor compliance with antiepileptic drugs, had a follow-up period of <12 months, and those with incomplete clinical information were excluded. Childbearing age was defined as between 15 and 49 years according to World Health Organization (23).

The demographic data, epilepsy etiology, onset age, treatment duration before switching, baseline seizure frequency, antiepileptic drugs, electroencephalograms, and brain imaging studies were documented. The baseline seizure frequency was calculated as the mean number of seizures per month within 3 months before switching from VPA. The reasons for switching, pre-switching seizure condition, VPA dosage before switching and every month thereafter, and any adverse effects of the antiepileptic drug were also recorded.

The primary outcome was successful switching, which was defined as a 50% decrease in the daily VPA dosage (mg/kg/dose) at the last clinic visit compared with pre-switching VPA dosage. The secondary outcome was defined as a decrease in the frequency and severity of adverse effects, which were assessed according to the medical records of pediatric neurological and obstetrics-gynecology outpatient clinics. This study was approved by the Chang Gung Memorial Hospital Institutional Review Board (201701598B0).

## Results

Twenty-six female patients were enrolled, of whom two were excluded (one was not within childbearing age, and the other lacked clinical information). The median age was 22.5 years, and the median disease onset age was 9.5 years. Most of the women had juvenile myoclonic epilepsy (*n* =11), followed by unclassified epilepsy (*n* = 3), autoimmune encephalitis (*n* = 3), frontal lobe epilepsy (*n* = 2), temporal lobe epilepsy (*n* = 2), benign childhood epilepsy with centrotemporal spikes (*n* = 1), hypoxic-ischemic encephalopathy (*n* = 1), post-traumatic brain injury (*n* = 1). Thirteen patients (54.2%) had pharmacoresistant epilepsy. Seven patients (29.2%) had positive brain magnetic resonance imaging findings, of whom four had hippocampal sclerosis, two had brain atrophy, and one had a pineal gland cyst. Nineteenth patients (79.2%) presented with focal epileptic discharges on interictal electroencephalography, two were generalized, and three were negative (Table 1). Ten patients (42%) switched antiepileptic drugs because they were planning to become pregnant, eight (33%) due to poor seizure control, and six (25%) due to adverse effects. The median pre-switch treatment period was 9 years, and the patients took a median of three antiepileptic drugs before switching, mostly VPA, clonazepam, and clobazam. The median baseline seizure frequency before switching was 0.25 times per month, and most of them presented with generalized tonic-clonic seizures. Minor seizures included staring, myoclonic, and eye blinking. Twenty of the 24 patients (83.3%) were classified into the successful switching group, and the median duration to complete switching drugs in this group was 19.5 months (Table 2). The median pre-switch VPA dosages were 11.5 and 6 mg/kg/day in the successful and unsuccessful groups, respectively. The baseline seizure frequencies were 0.25 times and 1 time per month in these two groups, respectively. At the last clinical follow-up after switching, the median VPA dosages were 0 and 17.6 mg/kg/day in the successful and unsuccessful groups, respectively (Table 2). The median levetiracetam dosage at last clinical visit was 9.4 and 14.6 mg/kg/day on successful and unsuccessful group. And the maximum levetiracetam dosage in this two groups were 38.6 and 29.8 mg/kg/day, respectively.

**Table 1:** The characteristics and etiologies of the 24 childbearing age women with epilepsy.

	**Successful group (*n* = 20)**	**Unsuccessful group (*n* = 4)**	**Total (*n* = 24)**
**ETIOLOGY**			
Non-symptomatic epilepsy	15	4	19
Juvenile myoclonic epilepsy	8	3	11
BECTS	1	0	1
Frontal lobe epilepsy	2	0	2
Temporal lobe epilepsy	2	0	2
Unclassified epilepsy	2	1	3
Symptomatic epilepsy	5	0	5
Encephalitis	3	0	3
HIE	1	0	1
Traumatic brain injury	1	0	1
Onset age (median, years)	8.5	11	9.5
EEG (focality, %)	79.2%	75%	79.2%
MRI (abnormal, %)	29.2%	25%	29.2%
Pharmacoresistant (%)	45%	100%	54.2%

*EEG, electroencephalogram; MRI, magnetic resonance imaging; BECTS, benign childhood epilepsy with centrotemporal spikes; HIE, hypoxic-ischemic encephalopathy*.

*p < 0.05.

**Table 2:** The switching efficacy of valproic acid to levetiracetam in 24 childbearing age women with epilepsy.

	**Successful group (*n* = 20)**	**Unsuccessful group (*n* = 4)**	**Total (*n* = 24)**
Switching duration (months)	19.5	16.5	18
VPA median dosage (mg/kg/day)			
Pre-switch (median)	11.5	6	11
Post-switch (median)	0	17.6	0
Seizure frequency (times per month)			
Pre-switch (median)	0.25	1	0.25
Post-switch (median)	0	0	0
Adverse effect of VPA (positive rate)			
Pre-switch	63.2%	60%	62.5%
Post-switch	0%	60%	12.5%

*VPA, valproic acid*.

*p < 0.05.

**Figure 1:**
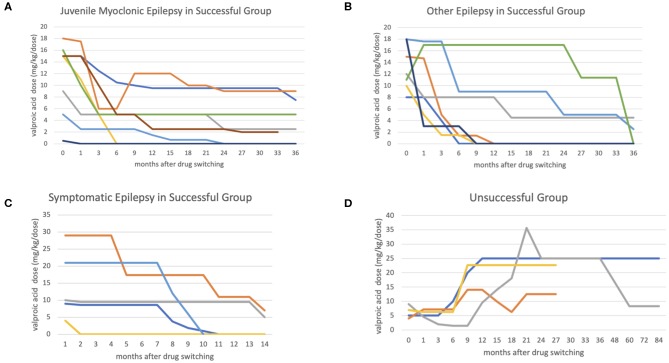
The figures show the antiepileptic drug switching time-dosage graph. The x-axis represents months after switching, and the y-axis represents valproic acid (VPA) dosage (mg/kg/day). In the successful group, most cases achieved a low VPA dosage (below 10 mg/kg/day) in 6 months in non-symptomatic epilepsy group **(A,B)**, comparing with symptomatic epileptic group **(C)**. In the unsuccessful group **(D)**, the VPA was added back at median 8 months after switching.

There were no major seizure attacks in the last 3 months in either group at last clinical follow-up, except in one of the cases with juvenile myoclonic epilepsy. The median switching duration was 19.5 months, and the VPA dose could be reduced to below 10 mg/kg/dose in a median of 6 months in the successful group (Table 2 and Figure 1).

The VPA adverse effect rates were similar in the two group (63.2 vs. 60%) before switching, most of which were alopecia, irregular menstruation, hand tremors, menorrhagia, and polycystic ovaries syndrome. The adverse effect rate reduced to 0% in the successful group, but was still 60% in the unsuccessful group (Table 2). In the successful group, it took a median 6 months to reach a stationary VPA dosage. In the unsuccessful group the seizures were poorly controlled, and VPA was added back in a median of 8 months. All four patients in the unsuccessful group needed a high VPA dosage to achieve seizure control initially, and one could be tapered VPA dosage at last clinical follow-up (Figure 1). The median VPA dosage in the unsuccessful group was 6 mg/kg/day, and the median seizure frequency was 1 time per month, which were not different to the successful group (Table 2). The only significant difference between the two group was the pharmacoresistance rate (45 and 100% in the successful and unsuccessful groups, respectively) (Table 1).

At the last clinic visit, four (17%) patients did not take any antiepileptic drugs, seven (29%) received levetiracetam monotherapy, 11 (46%) received VPA and levetiracetam polytherapy, and two (8%) received levetiracetam add other antiepileptic drugs. Three successful cases had already delivered children, the oldest of whom was 5 years old. All of the children had normal development and cognitive function, and none had major congenital malformations or development delays.

## Discussion

This is the first real-world study to investigate the efficacy and safety of switching from the high-risk teratogenic antiepileptic drug VPA to the new low-risk teratogenic drug levetiracetam. The high successful switching rate (83.3%) suggested that a switching strategy may be possible in most WWE. The teratogenic rate of levetiracetam has been reported to range from 1.6 to 2.4% in different studies (19), which is similar to a normal population without epilepsy (18). In the patients in the successful group with residual VPA treatment, the dosages were all below 700 mg/day, the median dosage was 4 mg/kg/day, and the highest dose was 11 mg/kg/day. Tomson et al. reported a teratogenic rate of 5.6% when the VPA dosage was below 700 mg/day (17). In our study, both the successful and unsuccessful groups achieved good seizure control after switching antiepileptic drugs. The VPA dose could be reduced to below 10 mg/kg/dose in a median of 6 months, and the median switching duration was 19.5 months. This information may be useful for clinicians when discussing with patients how early to start planning pregnancy. The major congenital malformation rate has been reported to be 5.6% with a VPA dosage below 700 mg/day, but up to 10.4% when the VPA dosage is above 1,000 mg/day. In addition, the major congenital malformation rate has been reported to be around 2.8% in children of untreated WWE, and 2.2% in the offspring of mothers without epilepsy (18). However, in the unsuccessful group in this study, the seizures became poorly controlled a few months after switching. Increasing the dose of levetiracetam could not achieve seizure control in these cases, and they needed VPA to be added back. Moreover, a higher VPA dosage compared to that before switching was needed to achieve seizure control. According to previous case reports of three and six patients, levetiracetam may aggravate myoclonic and absence seizures (24, 25). Other studies have reported more staring and generalized tonic-clonic seizures. In addition, switching antiepileptic drugs in previously well-controlled patients may lead to breakthrough seizures. Wang et al. performed a matched case cohort study in 2013, and found that the major predictive factor of post-switching seizure condition was the reason for switching. In the seizure-free on their old drug group, five (21.7%) patients had seizure recurrence after switching the drug compared to two of 46 (4.3%) of the matched controls. In addition, in the having seizures on their old drug group, six (30%) patients entered remission after switching the drug, compared to eight of 40 (20%) matched controls (26). We did not observe these findings in the current study, which may be due to the small number of cases. The percentages of those switching antiepileptic drugs due to poor seizure control in the successful and unsuccessful groups were 30 and 50%, respectively. Patients should be informed of these risks when discussing switching antiepileptic drugs. There were no significant differences in etiology, electroencephalograms, and magnetic resonance imaging findings between the successful and unsuccessful groups in this study, although the pharmacoresistance rate was higher in the unsuccessful group (45 vs. 100%). However, the reason for this finding is unclear, due to the small number of patients in the unsuccessful group.

Tomson et al. concluded that switching or discontinuing drugs during pregnancy was dangerous and associated with poor seizure control (2). Thus, careful planning for pregnancy, including seizure-free status and using low-risk teratogenic antiepileptic drugs is the most important strategy to achieve a safe pregnancy both for the mother and fetus, and avoid prematurity, major congenital malformations, and behavioral and cognitive disorders (3). Our observations suggested when to start planning for pregnancy and the successful switching rate. A higher successful switching rate may achieved with a longer switching time, and the minimum duration should be 2 years in our experience. We also observed that switching from VPA to levetiracetam may lead to poor seizure control in some cases initially, and the patients should be informed about this before switching. Switching could decrease the adverse effects of VPA in all cases with a lower VPA dosage in this study. The most common adverse effects were alopecia, acne, irregular menstruation, menorrhagia, hand tremors, and polycystic ovaries syndrome.

Since its first marketing as an antiepileptic drug in 1967 in France, VPA has become established worldwide as one of the most widely used antiepileptic drugs in the treatment of both generalized and focal seizures (27). Pre and post-synaptic effects of VPA depend on a very broad spectrum of actions, including regulation of ionic currents and facilitation ofγ-aminobutyric acidergic activities over glutamatergic transmission. As a result, VPA indirectly modulates neurotransmitter release and strengthens the threshold for seizure activity (28). Nevertheless, VPA have many adverse effects that require vigilance during the chronic treatment (27). The mechanisms of VPA related teratogenic effects were complicated, including folic acid deficiency, increased fetal oxidative stress, and change in gene expression by inhibition of histone deacetylase (HDAC) (29, 30). Folic acid supplementation and reducing VPA dosage during pregnancy could decrease fetal teratogenic rate and increase cognitive function (13).

There are three limitations to this study. First, this was a retrospective study with a limited number of cases. Second, most of the epileptic patients had juvenile myoclonic epilepsy, which may not represent all types of epilepsy. Third, in some cases, we added other low-risk teratogenic antiepileptic drugs to achieve seizure control after the switching had been performed. As polytherapy may also increase the risk of major congenital malformations, further studies are needed to investigate whether high-dose VPA monotherapy or low-dose VPA polytherapy combined with low-risk teratogenic antiepileptic drugs is preferable. Tomson et al. reported that most congenital malformations were dependent on the dose of VPA, regardless of monotherapy or polytherapy (17). Thus, using add-on levetiracetam with a lower VPA dosage may be a reasonable choice.

## Conclusion

Our experience supports switching from VPA to levetiracetam in WWE of childbearing age as an effective strategy to lower the teratogenic rate and adverse effects. Switching should be started early, at least 2 years before pregnancy, to achieve successful switching. Further prospective and case-controlled studies with larger sample sizes are necessary to elucidate the most effective and safe strategy for switching from VPA in WWE of childbearing age.

## Data Availability Statement

All datasets generated for this study are included in the article/Supplementary Material.

## Ethics Statement

This study was approved by the Chang Gung Memorial Hospital Institutional Review Board (201701598B0).

## Author Contributions

This research was performed at Chang Gung Children's Hospital in Taoyuan, Taiwan. I-JC, H-SW, and Y-SW conceived the study and C-YK, Y-HL, J-JL, and S-YL participated in data collection. K-LL, H-SW, and M-YH participated in study design and coordination. K-LL, H-SW, P-CH, M-LC, and M-YH contributed the patients. C-YK and Y-HL drafted the manuscript and K-LL critically revised the manuscript for important intellectual content.

## Conflict of Interest

The authors declare that the research was conducted in the absence of any commercial or financial relationships that could be construed as a potential conflict of interest.
